# Autoimmune optic neuropathy as the differential diagnosis of neuromyelitis optica spectrum disorders

**DOI:** 10.5935/0004-2749.20220064

**Published:** 2025-02-11

**Authors:** Lorranne Bandoli, Frederico Moura

**Affiliations:** 1 Universidade Estadual de Campinas, Campinas, SP, Brazil; 2 Universidade de São Paulo, São Paulo, SP, Brazil

**Keywords:** Autoimmune optic neuropathy, Optic neuritis, Neuromyelitis optica, Spectrum disorder, Diffusion magnetic resonance imaging, Neuropatia óptica autoimune, Neurite óptica, Desordem do espectro, Neuromielite óptica, Imagem de difusão por ressonância magnética

## Abstract

Optic neuritis is an important cause of decreased vision due to inflammation of the optic nerve. In view of its complex etiology, a thorough clinical evaluation is essential. Autoimmune optic neuropathy, a rare form of optic neuritis, is associated with progressive, painless, and severe visual loss. Severity depends on the inflammatory and ischemic components of the condition. Autoimmune optic neuropathy is ideally diagnosed with autoimmune disease markers (usually elevated levels of antinuclear antibodies). The treatment is immunosuppression with high doses of corticosteroids. Corticoid dependence is a characteristic of autoimmune optic neuropathy. In this report, we describe a patient with autoimmune optic neuropathy and discuss the importance of laboratory parameters and magnetic resonance imaging findings in the diagnosis of the disease.

## Abstract (pt)

A Neurite óptica é uma importante causa de diminuição da visão devido à inflamação do nervo óptico. Por apresentar diversas etiologias faz-se necessário ampla investigação. A neuropatia óptica autoimune corresponde a uma doença rara que se manifesta com perda visual aguda, indolor e grave. A gravidade está associada a sua fisiopatogenia com componentes inflamatório e isquêmico. A positividade para marcadores de doenças autoimunes, mais comumente a elevação da titulação de anticorpos antinucleares, são fatores determinantes para o diagnóstico da neuropatia óptica autoimune. O tratamento é feito através de imunossupressão, com necessidade de altas doses de corticoide. Neste relato iremos descrever um paciente com neuropatia óptica autoimune. Discutiremos sobre a importância dos parâmetros laboratoriais e os achados de imagem da ressonância magnética para o diagnóstico.

## INTRODUCTION

Autoimmune optic neuropathy (AON) was first described in 1982 by Dutton and coworkers in their report of 3 cases of retrobulbar optic neuritis with sudden and painful visual loss in patients who tested positive for autoantibody autoimmune disease but showed no systemic autoimmune manifestations^(1)^.

Subsequently, Kuppersmith identified the clinical features that distinguish AON from optic neuritis with optic nerve involvement due to demyelinating disease, in which patients usually recover vision with corticotherapy (or without treatment), whereas patients with AON develop severe visual sequelae^(2)^.

Severe visual loss requires prompt action. Screening for autoimmune disorders is recommended because of the high corticosteroid dosages administered in the acute and maintenance phases. Usually, patients with AON require therapy with corticosteroid-sparing immunosuppressants^(2)^. In this report, we describe a patient with AON and discuss the importance of laboratory parameters and magnetic resonance imaging (MRI) findings in the diagnosis of the disease.

## CASE REPORT

**Figure 1:**
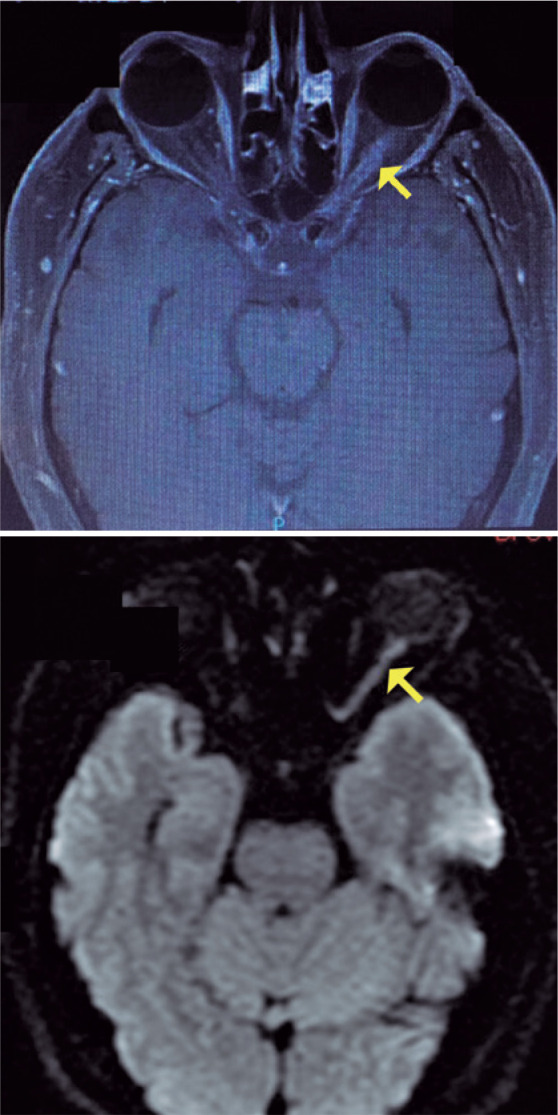
Magnetic resonance images of the brain and orbit showing enhancement of the left optic nerve.

A 58-year-old male patient was referred for neurophthalmic evaluation because of sudden and painless visual loss in the right eye over the preceding 2 months and worsening within a few days before referral. Approximately 1 year earlier, the patient experienced sudden and painless visual loss in the left eye. At the time, MRI scans of the brain and orbit revealed enhancement of the left optic nerve (Figure 1). On the basis of a tentative diagnosis of optic neuritis, the patient underwent intravenous methylprednisolone pulse therapy, but no visual improvement ensued.

**Figure 2:**
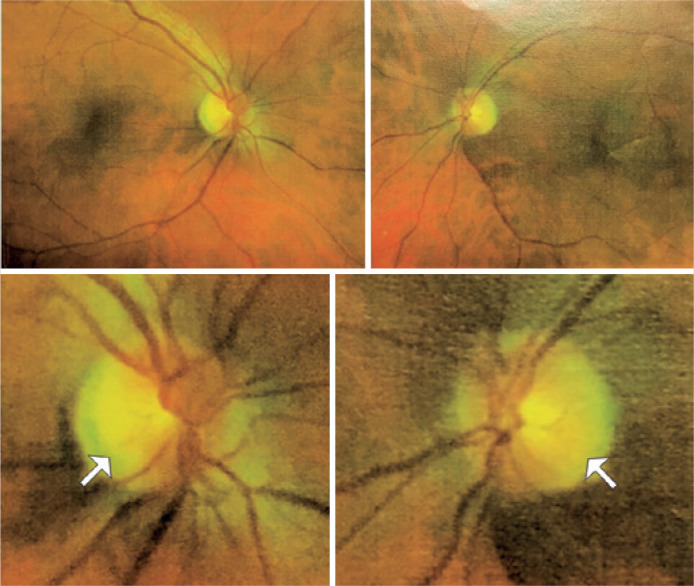
Fundoscopy image showing temporal papillary pallor in both eyes.

The patient’s visual acuity was 20/30 in the right eye and counting fingers in the nasal field in the left eye. Biomicroscopy and extrinsic ocular motility findings were normal, but a relative afferent pupillary defect of 3+ was observed in the left eye. Fundoscopy revealed temporal papillary pallor in both eyes (Figure 2).

**Figure 3:**
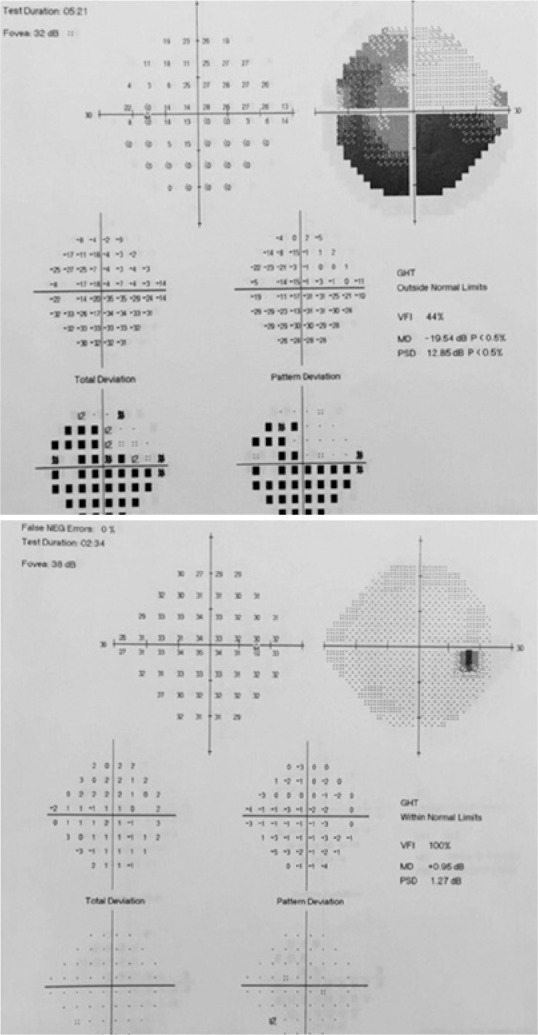
Standard automated perimetry findings for the first episode involving the left eye, including inferior altitudinal and upper temporal defects.

Unfortunately, standard automated perimetry (SAP) findings were not available for the episode involving the right eye, but the SAP findings for the episode involving the left eye included lower altitudinal and upper temporal defects (Figure 3).

**Figure 4:**
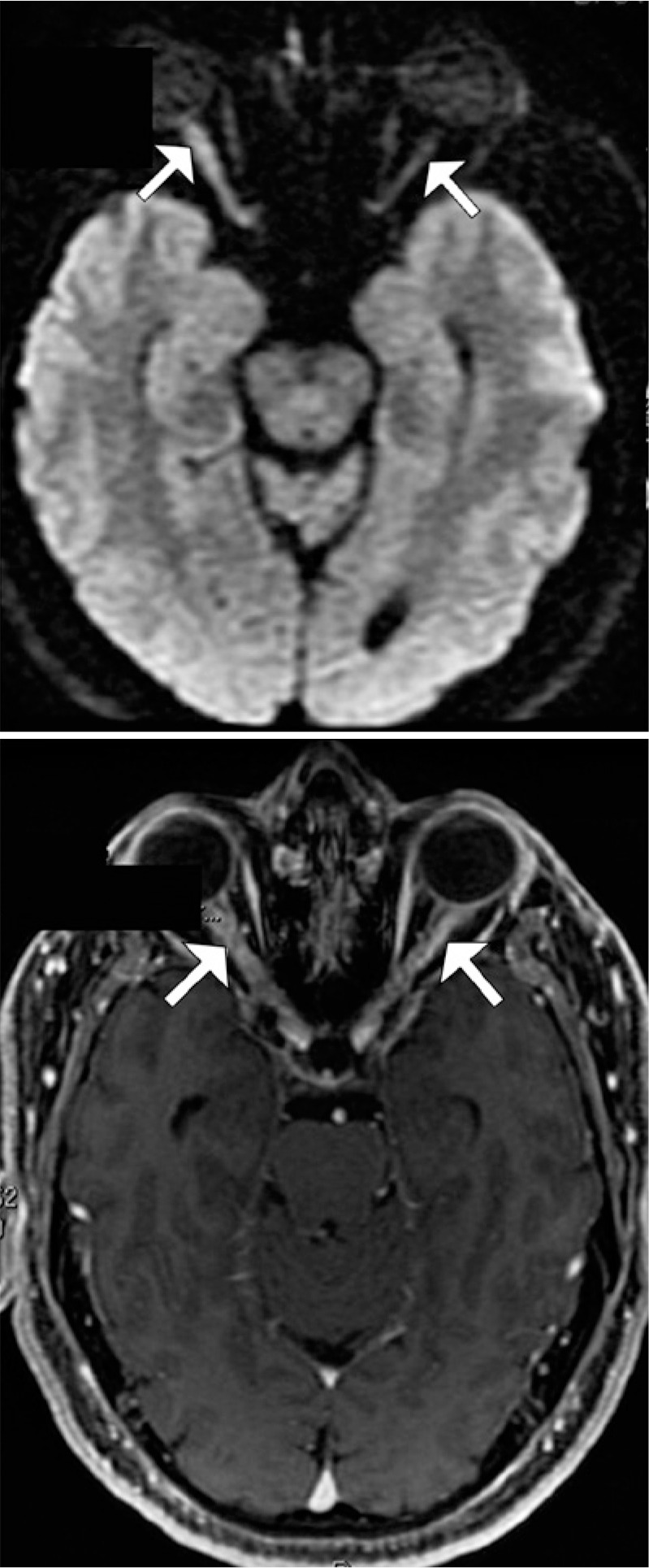
T2-weighted magnetic resonance images showing hyperintense thickening in both optic nerves that is associated with mild diffusion restriction.

T2-weighted MRI revealed hyperintense thickening in both optic nerves that was associated with post-contrast enhancement and mild diffusion restriction, which suggested concomitant inflammatory and ischemic processes in both eyes (Figure 4).

Under the suspicion of atypical optic neuritis, cerebrospinal fluid was sampled, and serological and inflammatory laboratory tests and assays for anti-AQP4 and anti-MOG antibodies were performed. The assistant neurologist ordered the following tests: antibody analyses (antinuclear antibodies, rheumatoid factor, anticardiolipin, anti-DNA, anti-beta2 glycoprotein, tissue anti-transglutaminase IGA and IGG, anti-endomisio IGA, and antineutrophil cytoplasmic antibody) and infection screening, including human immunodeficiency virus, syphilis, hepatitis B and C, herpes, cytomegalovirus, and toxoplasmosis. The LCR included the immunoglobulin index and oligoclonal bands. Aquaporine 4 antibody (CBA method) and anti-MOG (CBA method) were also tested before corticotherapy and only in the serum. The test results were within the normal ranges, except for the high fine speckled pattern ANA (antinuclear antibodies) titer (1:640). Skin biopsy was not performed to identify deposits of immune complexes.

Our diagnostic hypothesis was AON based on the severe, painless bilateral visual loss associated with the high ANA titer, findings of inflammatory and ischemic optic neuropathy on MRI, and the absence of systemic collagen disease. A second pulse therapy with intermittent immunoglobulin infusion was administered. The patient was followed up by the neurologist.

## DISCUSSION

AON differs pathophysiologically from demyelinating optic neuritis. In AON, the mechanism of the optic nerve injury has both inflammatory and ischemic components. In histopathological studies of optic nerve tissue, Riedel and colleagues observed chronic non-granulomatous perivascular inflammation^(4)^. These processes of vasculopathy and inflammation are limited to the optic nerve because no systemic disease is associated with the condition^(2-4)^.

In the original autoimune optic neuropathy report by Dutton et al, visual loss was associated with retrobulbar pain in all their three patients. However, painless visual loss has already been reported^(5)^, as well as in our patient. The absence of ocular pain corroborates the proposed ischemic component for the pathophysiology of autoimune optic neuropathy.

Owing to the dual mechanism of injury, visual loss is potentially severe in patients with AON. It tends to be mild initially and, in the absence of treatment, progresses over weeks or months. The optic nerve may be normal or show optic disc edema. Optic nerve atrophy is a late sign of involvement^(2,4)^.

The most important laboratory finding in AON is high ANA titer. However, ANA positivity is not AON specific. ANA positivity has been observed in several diseases and even in healthy individuals^(3)^. A multicenter study by Tan et al. on the frequency of ANA positivity and titration in healthy individuals showed that 31.7% of healthy people have titers of 1/40, while titers of 1/320 and higher (as in the present case) are found in only 3.3%^(6)^. ANA positivity in healthy people (i.e., false positive) occurs especially in women^(7)^. However, no reported case of AON presented an associated systemic autoimmune disease^(2)^.

Our patient had painless, bilateral, and recurrent visual loss associated with the lack of visual recovery after treatment with corticosteroids. These characteristics are suggestive of optic neuritis in neuromyelitis optica spectrum disorders (NMOSD). As anti-aquaporin 4 and anti-MOG were negative, our case was classified as NMOSD double negative. The latest international consensus^(8)^ that defined the diagnostic criteria defines NMOSD AQP4 negative as

1. at least 2 core clinical characteristics occurring as a result of one or more clinical attacks and meeting all the following requirements:

a. At least 1 core clinical characteristic must be optic neuritis, acute myelitis with LETM, or area postrema syndrome.

b. Dissemination in space (≥2 different core clinical characteristics).

c. Fulfillment of additional MRI requirements, as applicable.

2. negative tests for AQP4-IgG using the best available detection method, or testing unavailable; and

3. exclusion of alternative diagnoses.

As the diagnostic criteria require at least 2 core clinical characteristics (among optic neuritis, acute myelitis, area postrema syndrome, acute brainstem syndrome, acute diencephalic clinical syndrome, and symptomatic cerebral syndrome), our patient did not meet the criteria because he did not present any of the clinical signs described earlier, other than optic neuritis, during follow-up.

Another fact present in our case that suggests NMOSD is the presence of ANA, which occurred in 40% of patients with NMOSD, as shown in this study^(9)^. This high frequency of ANA is found in patients with NMO and anti-aquaporin 4-positive antibody^(9)^. In NMO AQP4-negative patients, this frequency is lower, as in the study of Sato et al. that showed ANA in only 15% of NMO AQP4-negative patients^(10)^.

For patients with double seronegative NMOSD, further research is needed to better elucidate the clinical and immunopathological features and to define whether AON should be considered as part of NMOSD. T h e finding of diffusion restriction on MRI suggests that visual loss was associated with ischemic injury and supports the notion of a mixed mechanism of inflammation and ischemia in AON^(11)^. However, we think that the presence of diffusion restriction on MRI helps to differentiate demyelinating optic neuritis from other inflammatory optic neuropathies such as NMO and AON.

In conclusion, in patients with atypical optic neuritis with negative AQP4 and anti-MOG and positive ANA associated with diffusion restriction on MRI restriction, AON is a likely diagnosis.
